# Evidence for content-dependent timing of real-life events during COVID-19 crisis

**DOI:** 10.1038/s41598-022-13076-6

**Published:** 2022-06-02

**Authors:** Keren Taub, Dekel Abeles, Shlomit Yuval-Greenberg

**Affiliations:** 1 Sagol School on Neuroscience, Tel-Aviv University, Tel Aviv-Yafo, Israel; 2 School of Psychological Sciences, Tel-Aviv University, Tel Aviv-Yafo, Israel

**Keywords:** Neuroscience, Cognitive neuroscience, Perception

## Abstract

How do people estimate the time of past events? A prominent hypothesis suggests that there are multiple timing systems which operate in parallel, depending on circumstances. However, quantitative evidence supporting this hypothesis focused solely on short time-scales (seconds to minutes) and lab-produced events. Furthermore, these studies typically examined the effect of the circumstance and the psychological state of the participant rather than the content of the timed events. Here, we provide, for the first time, support for multiple content-based timing systems when estimating the time of real-life events over long time-scales. The study was conducted during the COVID-19 crisis, which provided a rare opportunity to examine real-life time perception when many were exposed to similar meaningful events. Participants (N = 468) were asked to retrospectively estimate the time that has passed since prominent events, that were either related or unrelated to the pandemic. Results showed an overall time-inflation, which was decreased for events related to the pandemic. This indicates that long-term subjective timing of real-life events exists in multiple systems, which are affected not only by circumstances, but also by content.

## Introduction

"We all agree that March was six years long and April has lasted six minutes, right? … Genuinely baffled by how elastic time is right now, but more by how we seem to be experiencing it in similar ways."

- Louis Peitzman, American actor (Twitter, April 29th, 2020 amid the COVID-19 outbreak).

The distorted perception of time described in the citation above, is not unique to the COVID-19 outbreak: many individuals report experiencing time distortion sporadically throughout their lives. The idea that the passage of time, as it is subjectively perceived by an individual, may differ from objective time, has already been suggested by William James in the nineteenth century. James raised the hypothesis that when estimating time in retrospect, time is perceived to last longer if much has happened during its course, and shorter if only little has happened^1^. The COVID-19 pandemic created a rare situation in which a large number of individuals were affected by the same significant events, with both global and personal implications, at the same objective time. This unique situation was an opportunity to examine factors affecting subjective time estimations. Specifically, how accurate are estimations of the amount of time that has passed since the occurrence of known events, what influences these estimations and how they are affected (or not) by psychological factors.

Early theories of time perception postulated that the ability to estimate the passage of time is based on a single timing mechanism—a pacemaker-accumulator internal clock^2^. It was further suggested that various factors such as age, cognitive factors and emotional valence may change the pace of this internal clock, biasing it to be either faster or slower than the objective time, which could lead to temporal distortions^3^. Other theories, suggested that time estimation is mediated, not by a single mechanism, but by multiple neural systems^4^. According to this hypothesis, the different timing systems could function independently and simultaneously. Evidence for this view comes from studies showing that different tasks activate different timing mechanisms that exist simultaneously in the same perceiver^5^. It is an open question whether these multiple systems could also take part in temporal processing of real-life events. That is, whether multiple events that occurred in close temporal proximity, as often happens in everyday life, could be perceived as occurring at different times, reflecting separate timing systems.

Time estimation can be examined either prospectively, when participants are encouraged to attend to temporal information that they know that they will be asked about later, or retrospectively, when participants are unaware of the timing task until after the events have already been presented. Findings show that retrospective estimations of time tend to be less accurate and more variable than prospective estimations^6^. Studies of retrospective timing have typically presented participants with stimuli lasting for certain durations and then asked them to estimate these durations in retrospect^7–9^. Another, more unique, type of retrospective time estimation task is the *judgment of the passage of time*—when participants are asked to assess how much time has passed since a certain event and until present time. In a series of studies, Flaherty^10,11^ used qualitative methods to examine biases in retrospective judgements of the passage of time in everyday life. According to Flaherty, *synchronicity*, a match between objective time and subjective time, occurs in circumstances that are relatively unproblematic, not involving strong emotions and that are neither very simple nor very complex. In contrast, *temporal compression*, that is, the experience of time moving faster than usual, occurs when conscious information processing is low. *Protracted duration*, or time inflation, occurs when circumstances are either extremely eventful and include high levels of complexity, or when they are extremely uneventful and non-complex. Flaherty suggested that the subjective involvement in these unusual circumstances creates a greater density of experience per standard temporal unit, causing the sensation of time inflation. In his studies, Flaherty^10,11^, used a qualitative approach, which had several limitations. First, these studies focused on individuals who were involved in exceptional circumstances, and consequently conclusions were limited in their generalizability. Second, the studies used introspection: when asked to introspect people may have been inaccurate due to embarrassment or forgetfulness. Last, these methods were unsuitable for comparing objective and subjective time, because the information on objective time and its deviation from the subjective report was usually unobtainable.

Time estimation distortions were also examined in lab-based studies that provided quantitative measures. In these studies, focusing mostly on prospective duration estimations rather than retrospective passage of time estimations, participants are typically requested to estimate the duration of short time-intervals, typically lasting between a few hundred milliseconds to a few minutes^12^. Findings suggested that factors such as individual traits, psychological state and physiological arousal modulate time perception over these short periods of times^13,14^. However, whereas lab-based approaches can provide accurate assessment of errors in subjective timings, they typically measure only short time periods^15,16^, and therefore are less suitable for studying retrospective perception of the passage of time in long-scale durations and in every-day life. In this study, conducted at the onset of the COVID-19 outbreak, we utilized a novel approach to quantitively study retrospective estimation of the passage of time over long intervals (days to months) and in real-life events.

The outbreak of COVID-19 affected almost everyone around the globe, providing, along its devastating consequences, a rare research opportunity on the effects of these unique events on a variety of people. This was a unique opportunity to compare subjective and objective time, using a non-lab-based quantitative approach^17^. A study conducted in the UK during the first lockdown reported that time distortions were common among participants. The findings showed that time distortion was affected by demographic factors such as age and social satisfaction^18^. In another study on time perception conducted in Italy during the first COVID-19 lockdown, participants’ introspective reports reflected that they have experienced time inflation^19^. These studies relied on qualitative subjective introspections of an experience of time distortions.

In this pre-registered research, conducted during the first COVID-9 lockdown (in April 2020), we quantified errors in estimation of the passage of time by comparing subjective estimations of prominent events, with their objective times. Our goal was to examine the hypothesis that, when estimating time on long scales (of days to months), real-life events of different contents and contexts would activate different timing systems. Specifically, we predicted that the subjective perception of the passage of time would be affected not only by its objective timing, but also by its specific characteristics (i.e. whether its related to the COVID-19 or not). To this end, we investigated errors in subjective timing of events that were either related or unrelated to the COVID-19 outbreak. In addition, we measured psychological factors (anxiety, stress and COVID-related stress) which were previously shown to modulate time perception^14,20^, and demographic factors that could affect the load of events in one's life at times of lockdown, and therefore could affect the perception of time. The findings supported our main hypothesis by demonstrating, for the first time, the existence of multiple content-related timing systems in long-scale estimation of the passage of time. Our results also show, counter to our hypothesis, that errors in time estimation were not explained by psychological and demographic factors.

## Methods

### Participants

We recruited 598 Israeli adult participants for an online study during the COVID-19 crisis. Following pre-registered exclusions (https://osf.io/3kzve/), 468 participants were included in the study (244 females, ages 18–96, M: 41.05, SD: 14.60). Most (412) participants were recruited online through the Israeli internet surveys company Panel4All and received a small payment. The rest of the participants (56) were volunteers who responded to a post on our lab Facebook page.

Sample size was based on power analysis (using Gpower 3.1), as was declared prior to data collection in a preregistration document. Our estimated parameters for linear multiple regression were a small effect size of 0.02 R^2^ change, with significance level of 0.05 and power of 0.8. Power estimation indicated a required sample size of N = 395. The total number of participants (468) acceded this number.

Participation was completely anonymous, no identifiable details were collected, and participants signed their informed consent by pressing a key at the beginning of the questionnaire. The study was approved by the ethical committee of Tel-Aviv University (research proposal no. 0001412-1). The experiment was performed in accordance with the relevant guidelines and regulations of Tel-Aviv University and with international guidelines for performing non-medical research in humans.

### Constructing a set of familiar current events

Prior to the main experiment we have conducted a preliminary study for establishing a set of current events to be used in the main experiment. The purpose of this study was to ensure that all the included events were highly recognizable among general Israeli population. Fifty-one participants were presented with a list of 43 current events and were asked to report whether they are familiar with them or not. Only events that were familiar to at least 80% of the participants in the preliminary study were included in the main study. This resulted in the inclusion of 32 events, 20 of them related to the COVID crisis (“COVID events”) and the rest unrelated (“non-COVID events”). Each of the two sets included events of both high and low emotional valance and events that had a direct impact on the individual’s lives and others that had less impact (see full list on Supplementary Material S1).

Out of these 32 events, we were able to arrange 22 events in matching pairs of 11 COVID and 11 non-COVID events, such that the pairs of events occurred at no more than five days apart. One COVID event remained out of this pairing because it had no matching event among the non-COVID set. These pairs were balanced so that in some of them the COVID event was less recent and in others the non-COVID was less recent. We chose to pair the events in a way that would minimize the difference in time across all pairs (the average difference between the pairs of events was 0.366 days, SD = 2.46 days). The outcome was that out of 11 pairs, in one pair both events occurred on the same date, in five pairs the COVID event was less recent (but by no more than 5 days), and in the rest, the non-COVID event was less recent (again, by no more than 5 days). No other arrangement would have led to a smaller mean time difference between the events. See Supplementary Material S1 for the full set of events and their pairings.

### Procedure

After giving consent, participants were presented with a few personal questions regarding age, gender, marital status, parenthood, occupation and employment. This was followed by a few questions for determining how much they were affected by the COVID-19 crisis. These questions focused on health condition, smoking habits, number of people living in the same household (and their ages), whether they are considered to be at risk for the virus, and how the outbreak affected their occupation and income.

This was followed by the first stage of the main study, in which participants were presented with the list of 32 current events (see above). COVID and non-COVID events were presented in a mixed and non-chronological order, and this order was fixed between participants. Following the description of each event, participants were asked to indicate whether they are familiar with it or not. If they were familiar with the event, they were further asked to indicate on scale of 0 to 90 days (using a moving slider), how much time they estimate to have passed since the occurrence of this event.

In the second stage of the study, participants were presented with the 11 pairs of COVID and non-COVID events (see above), and were asked to determine which of the two events was less recent, and then estimate how many days have passed between these events. All the events included in this stage of the study, were included also in the first stage.

Following the main two stages of the study, participants completed three psychological questionnaires: the Hebrew version of the Perceived Stress Scale, with four levels^21,22^, the Hebrew version of the Generalized Anxiety Disorder 7-items (GAD-7) scale, developed by Spitzer et al.^23^, and the COVID-19 threat scale^24,25^. In addition, participants performed a short financial decision-making task, which we opted to leave out of the present study. Analysis of this task is reported in Supplementary material S2, consistent with the pre-registration document.

### Exclusion criteria

Events that were not recognized by the participants were excluded from analysis. If an unfamiliar event was part of the 22 matching events, its pair was also excluded. Participants were discarded from analysis if they marked that they are familiar with five events or more without adjusting the slider from its initial position, or if they did not recognize more than 20% of the events.

### Analysis

Data was collected using the web-based software Qualtrics.com. All retrieved data was process using MATLAB R2021b. Graphs were generated using MATLAB R2021b and processed through Adobe Illustrator 2022. Statistical analyses were performed using IBM SPSS Statistics 27. Multiple comparisons were corrected using false discovery rate (FDR) correction^26^. All the analyses performed in this study were described in the pre-registration document. Two of these analyses are included in Supplementary material S2 and the rest are in the main manuscript.

## Results

### Subjective vs. objective timing of events

**Figure 1:**
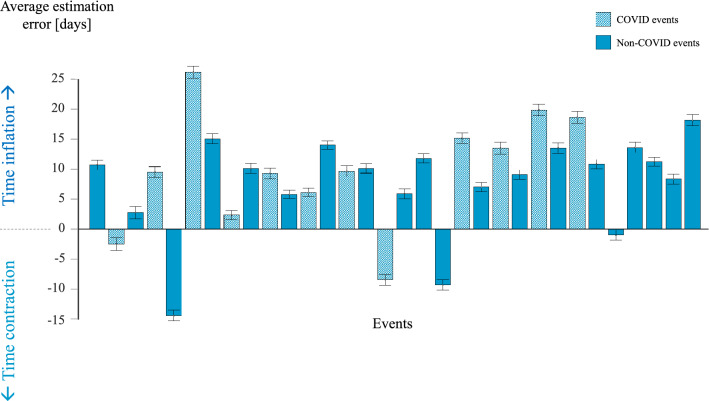
Average estimation errors for the 32 events included in the first stage of the experiment. Errors were calculated as subjective time estimations minus the objective times. Hence, positive values represent an inflated estimation and negative values represent contracted estimation, relative to the objective time.

**Figure 2:**
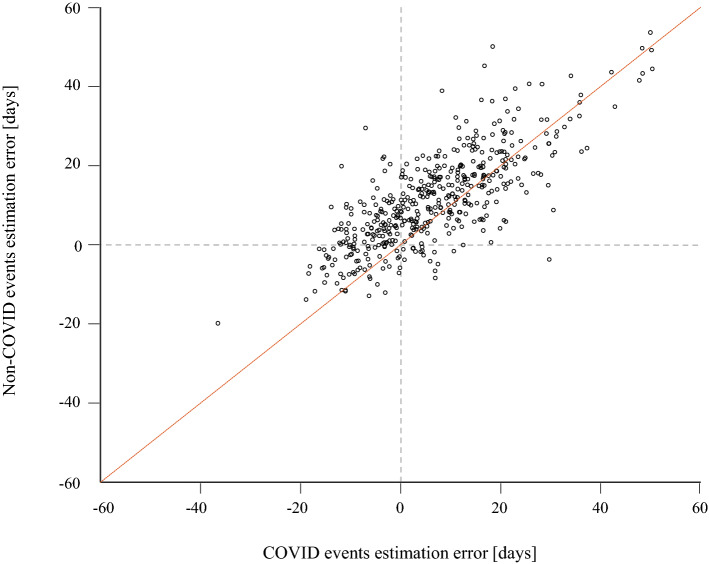
OVID and Non-COVID estimation errors. The scatter plot depicts the estimation errors of all participants (n = 469). The x-axis represents average estimation error of COVID events, and the y-axis represent the average estimation error of non-COVID events. The red line represents the equality line: indicating no difference in estimation error between COVID and Non-COVID events. The figure indicates that for both types of events, participants tended to over- rather than under-estimate time. Most participants are above the equality line, indicating that there was more over-estimation for non-COVID than for COVID events.

In the first stage of the study, participants were asked to estimate, on a scale of 0 to 90, how many days have elapsed since the occurrence of each of the 32 events. The subjective time estimations were averaged across all the included events for each participant. The estimation errors were calculated by subtracting the average objective times – the actual number of days that have elapsed between the event and the testing—from the average subjective time estimations. Averaging these errors across the events revealed that participants tended to estimate the time that has elapsed since an event as significantly longer than the actual time that had elapsed, indicating that subjective timing was inflated (subjective minus objective time: Mean = 8.548 days, SD = 1.789 days; errors compared with zero: t(467) = 15.686, *p* < 0.001; Figs. 1, 2). This analysis was performed on the full set of 32 events chosen based on the preliminary study. This set included 20 COVID events and 12 non-COVID events and therefore was unbalanced in this respect. To ensure that the findings were not modulated by this imbalance, we have conducted the same analysis on a balanced subset of 24 events (12 COVID and 12 non-COVID that were randomly picked out of the larger event set). This analysis revealed that subjective timing was inflated, as in the original analysis (subjective minus objective time: Mean = 9.465 days, SD = 11.829 days; errors compared with zero: t(467) = 17.310, *p* < 0.001).

**Figure 3:**
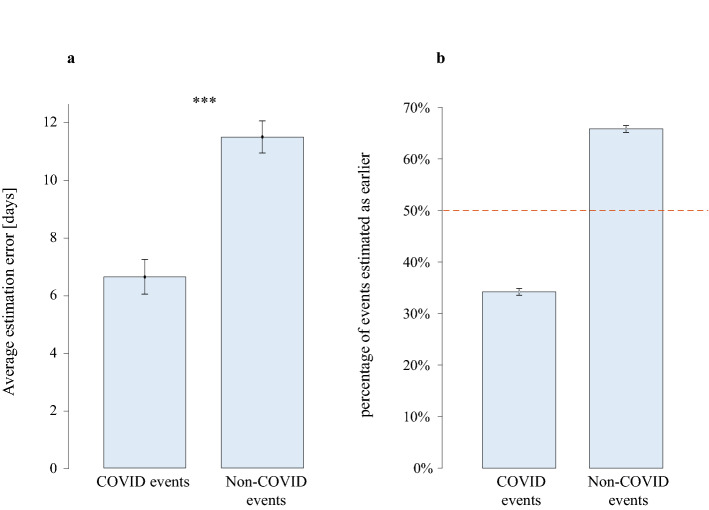
COVID vs. Non-COVID estimation errors for 11 pairs of events. (**a**) Average estimation errors based on the first stage of the experiment. While both types of events show time inflation, time estimation for the non-COVID events was significantly more inflated than for COVID events. (**b**) Percentage of COVID vs. non-COVID events estimated as less recent within their pair, based on the second stage of the experiment. Dashed line represents the percentage of the events that were less recent (very near 50% COVID and 50% non-COVID). Consistently with findings of the first stage, these results show that Non-COVID events were perceived to be less recent than their counterpart COVID events. ***p < 0.001.

Next, we compared subjective time estimations errors for events that were related to COVID-19 to those that were unrelated. For each of these sets of events (COVID/non-COVID) we calculated the time estimation error values (subjective minus objective time) and compared COVID and non-COVID events. Findings showed a significant difference between the two types of events, with COVID events experienced as more recent than the non-COVID events (time estimation error for COVID events: M = 6.629, SD = 13.055; time estimation error for non-COVID events: M = 11.482, SD = 12.011; COVID > non-COVID: t(467) = 12.234, *p* < 0.001; Fig. 3).

#### Which was less recent: COVID or non-COVID events?

In the second stage of the study participants were asked to determine for each of the 11 pairs of events, which event was less recent. Additionally, they were asked to estimate the number of days that have elapsed between the two events.

**Figure 4:**
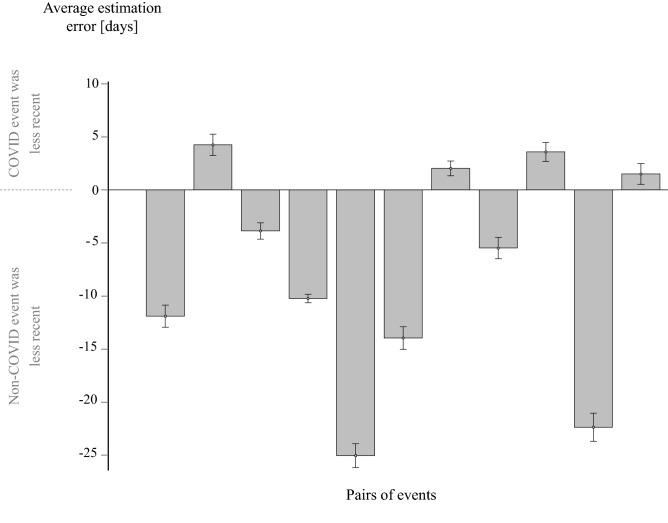
Average estimation errors of time passing between the COVID and the non-COVID event of each of the 11 pairs of events. Zero represent the correct time estimation. Positive values represent estimation errors that placed the COVID event as less recent than it was compared to the non-COVID event. Negative values represent estimation errors that placed the non-COVID event as less recent than it was compared to the COVID event. For most (7/11) pairs, errors indicated that COVID events were perceived as more recent than their counterpart non-COVID events.

Consistently with the previous analysis, results showed that participants were significantly more likely to perceive the COVID event as more recent than the non-COVID event. In 65.8% of the pairs on average (7.238 out of 11, SD = 14.04%), participants rated the non-COVID event as less recent than the COVID event and on only 34.2% they rated the opposite (binary t-test: t(467) = 24.353, *p* < 0.001; Fig. 3). Furthermore, we averaged the number of days that were estimated to have passed between each COVID and non-COVID event within each of the pairs. We then assigned a negative sign to each value that represented a pair for which the COVID event was estimated to be less recent than the non-COVID event. Thus, positive values of this measurement indicate that COVID events were estimated as less recent than non-COVID events, and vice versa for negative values. The average of these values across participants was -7.38 days (SD = 7.828). The negative average indicates that participants tended to estimate the non-COVID events as less recent than the COVID events. This value was significantly different than zero across participants (t(467) = 21.423, *p* < 0.001; Fig. 4).

#### Predicting subjective time estimation by demographic and psychological factors

In addition to the time estimation tests, we collected data on demographic factors such as age, occupation and living status, and on psychological factors such as stress, anxiety and COVID related threat, which was measured using the Hebrew-version of the COVID-19 threat scale^24,25^. We found significant correlations between time estimations (overall time estimation; time estimation for COVID events only; time estimation for non-COVID events only) and some of the demographic variables: age, age^2^, number of people living in the house and number of children under 18 living in the house. Age^2^ was included in order to allow the inspection of quadratic relations with age, as is a common practice in the field^27–29^. All other correlations with time estimations were found to be insignificant (See full data in Table 1).

**Table 1:** Pearson correlation coefficient between time estimation measurements and demographic and psychological factors.

	Age	Age^2^	Gender	Anxiety	Stress	COVID related threat	Change to occupation (N = 382)	High risk group (N = 437)	Num. of people in the house	Num. of people under 18	Num. of people over 60
Overall time estimation	−0.131*	−0.135*	0.022	0.071	0.022	−0.058	−0.061	−0.042	0.135*	0.152**	−0.009
COVID related events	−0.145**	−0.148**	0.027	0.065	0.033	−0.065	−0.064	−0.061	0.149**	0.147**	0.005
Non-COVID related events	−0.116*	−0.125*	0.033	0.050	0.004	−0.071	−0.073	−0.059	0.108	0.160**	−0.033
Age		0.984***	−0.025	0.007	−0.166**	−0.027	−0.070	0.503***	−0.260***	−0.134*	0.126*
Age^2^			−0.033	0.010	−0.161**	−0.007	−0.037	0.540***	−0.275***	−0.192***	0.168**
Gender				0.001	−0.006	0.088	0.040	−0.004	−0.041	−0.030	−0.025
Anxiety					0.646***	0.286***	0.060	0.108	0.015	−0.014	0.064
Stress						0.302***	0.146*	−0.013	0.064	0.017	0.031
COVID related threat							0.100	0.096	−0.015	−0.025	0.010
Change to occupation (N = 382)								0.026	−0.064	−0.175**	0.112
High risk group (N = 437)									−0.159**	−0.134**	0.125**
Num. of people in the house										0.691***	0.019
Num. of people under 18											−0.226***
Num. of people over 60											

*Change to occupation due to COVID-19:* only participants who reported either keeping their job or losing their job following the COVID-19 outbreak, were included in the sample. Participants who chose “other” as an answer for this question, were removed from the sample for this correlation analysis (N = 86). *High risk for COVID-19:* Participants who responded that they do not know whether they are at risk for COVID-19 or not, were removed from the sample for this correlation analysis (N = 31).

*p < 0.05, **p < 0.01, ***p < 0.001; all *p*-values were FDR corrected.

Furthermore, we conducted a hierarchical linear regression analysis to test the contribution of three of the demographic factors (gender, age and age^2^), and the three psychological factors (anxiety, stress, and COVID threat perception) on the prediction of the overall subjective time estimation (collapsed on all the events together). First, we tested a linear model including the three demographic factors as predictors. This model was insignificant (F(3,464) = 2.915, FDR corrected *p* = 0.238, R^2^ = 0.019). In addition, following FDR correction, none of these factors predicted subjective time estimation individually (age: β = 0.032, FDR corrected *p* = 0.1.052; age^2^: β = -0.166, FDR corrected *p* = 0.922; gender: β = 0.018, FDR corrected *p* = 0.981). Second, we added the three psychological factors (anxiety, stress and COVID threat perception) as predictors to the hierarchical model but none of them contributed significantly to the model (anxiety: FDR corrected *p* = 0.396, stress: FDR corrected *p* = 0.990, COVID threat scale: FDR corrected *p* = 0.443).

Similar results were obtained when constructing linear regression models to predict the subjective time estimation of COVID and non-COVID events separately. The subjective time estimation of COVID related events was not predictable by the three demographic factors when they were combined (F(3,464) = 3.563, FDR corrected *p* = 0.098, R^2^ = 0.023), and none of the predictors was found to be individually significant (age: β = 0.039, FDR corrected *p* = 0.881; age^2^: β = −0.186, FDR corrected *p* = 0.835; gender: β = 0.022, FDR corrected *p* = 0.886). The psychological factors did not contribute significantly when added to this model (anxiety: β = 0.067, FDR corrected *p* = 0.343; stress: β = 0.010, FDR corrected *p* = 0.964; COVID threat scale: β = −0.069, FDR corrected *p* = 0.490). Similar results obtained for the non-COVID events (when the three factors were combined: F(3,464) = 2.8, FDR corrected *p* = 0.28, R^2^ = 0.018; contribution of individual predictors: age: β = 0.210, FDR corrected *p* = 0.592; age^2^: β = −0.331, FDR corrected *p* = 0.483; gender: β = 0.027, FDR corrected *p* = 0.649; anxiety: β = 0.052, FDR corrected *p* = 0.448; stress: β = −0.014, FDR corrected *p* = 0.761; COVID threat scale: β = −0.071, FDR corrected *p* = 0.441).

The same analysis was conducted to predict the subjective estimation of the average time that had passed between the paired COVID and non-COVID events. Again, following FDR correction, we found no contribution to the model including the three demographic factors (F(3,464) = 3.264, FDR corrected *p* = 0.147, *R*^2^ = 0.021), and the predictors were not found to be individually significant (age: β = −0.111, FDR corrected *p* = 0.783; age^2^: β = −0.017, FDR corrected *p* = 0.948; gender: β = 0.062, FDR corrected *p* = 0.616). Same as above, the psychological factors did not contribute significantly when added to this model (anxiety: β = 0.029, FDR corrected *p* = 0.738; stress: β = 0.044, FDR corrected *p* = 0.606; COVID threat scale: β = 0.055, FDR corrected *p* = 0.562).

## Discussion

In this study we show evidence for a distorted estimation of the passage of time during the first lockdown of the COVID-19 outbreak. The findings indicate different distortion for events that were related or unrelated to the pandemic: while in general, participants tended to experience an inflation of subjective time, this effect was smaller for events that were related to the pandemic than for those that were unrelated. In other words, events that were related to the pandemic were perceived to have occurred nearer to present time and closer to their actual time, than other events. Despite previous account linking psychological factors to time perception^14,20^, anxiety, stress and demographic factors (except age) were not found to correlate with the ability to accurately estimate the passage of time.

The finding that the experience of time inflation was smaller for events that were related to the pandemic, highlights the flexibility of subjective time—not only the nature of the circumstances under which time is estimated, but also the contents of the events that are estimated, determine their subjective timing. These results are consistent with a prevailing view, that there is no unique, homogenous experience of time, but multiple experiences of time, depending on context^30–32^. These findings suggest that multiple systems can lead to multiple dimensions of time perception even on long intervals of time, indicating that time perception is affected by the characteristics of the event. That is, while the experiment included events that occurred very close to each other in time, participants not only experienced distortion in the subjective perception of the time that had elapsed, but they also experienced different distortions for different types of events, reflecting the different timing systems that were activated by these events.

There are a few possible explanations as to why COVID-related events activated a different time estimation system than non-COVID related events. First, COVID-related events were part of an ongoing situation, presumably leading to their categorization with previous COVID-related events. This binding together, or “temporal Gestalt”, of the COVID-related events, may have eventually led to the subjective experience that these events were closer to present time than the unrelated events. A second interpretation is based on the *strength theory*^33^, suggesting that events that leave stronger memory traces, i.e. traces that are more vivid, detailed or suffer less interference, are perceived as more recent^34^. COVID-related events, which were prone to leave stronger traces as they were more unique^35,36^, may have been remembered as more recent than events that were unrelated to the pandemic. When constructing the set of events, we have made an effort to balance the two sets in terms of their emotional valance, personal relevance and novelty. However, these factors were not explicitly measured and, therefore, we cannot rule out that they have also contributed to differences in the way events of the two sets were retrieved. A third explanation derives from the *construal level theory*, which suggests that various psychological distances, such as the perceived distances in time and space, are cognitively related to each other and that proximity is associated with more concrete and detailed representations^37^. Following this theory, it is possible that during lockdown, participants perceived events that are related to the pandemic as more relevant or important to them, i.e. “closer” in psychological distance, and therefore encoded them in more detail. This may have led to their perception as closer in time. Vice versa, events that were unrelated to the pandemic and therefore less relevant to the individual and more psychologically distant, were encoded with less details and therefore perceived as more distant in time^37,38^. One last explanation is based on the *context overlap theory,* which suggests that the amount of contextual overlap between encoding and recall is correlated with how recent the event is perceived, with greater shared context leading to a more recent perception of the events^39,40^. It could be hypothesized that COVID-related events were perceived as more recent compared with events that were unrelated to COVID, since the time of retrieval was during a COVID-induced lockdown. Therefore, the context of life at the time of retrieval was related to the content of these events, making COVID-related events seem more recent compared with other events.

An important aspect of this research is the time in which it was taken place. Our study was conducted in April 2020, during the first COVID-19 lockdown in Israel. This was a time of extreme stress, anxiety and uncertainty for many people around the world, including Israel. The lives of many have changed dramatically and basic human rights were violated by COVID-safety regulations^41^. Importantly, this was a highly eventful period of time in which current events and personal events intermingled. According to the protracted duration phenomenon by Flaherty^10,11^, when circumstances are highly eventful, very complex or extremely different than everyday life, subjective time is inflated. Our finding of a general time inflation during the COVID crisis is consistent with this hypothesis, and is most probably its first quantitative support. However, a limitation of this overall analysis of events is that it allows only a comparison of subjective and objective time estimation, but does not prove the link of the observed effect to the COVID-crisis. With the present findings collected during the onset of the COVID outbreak, we can only hypothesize that the observed subjective time distortions are related to the unique characteristics of this specific time period. Confirming this hypothesis would not be trivial and is arguably even impossible, as different periods of time come with different personal and public emotional impacts and therefore a “baseline” time period cannot be easily defined, if at all. We believe that this is one demonstration of the inherent disadvantages of studies that are based on real-world occurrences and conducted in an ecological setting. However, studies such as this one could offer a unique contribution that, in this case, we believe to surpass the limitations.

Despite extensive research demonstrating the effects of emotion on time estimation^20,42–44^, in the present study we found no evidence that time inflation was affected by self-reported levels of anxiety or stress or by demographic factors that could have elevated them (e.g. being at risk for COVID-19). A possible explanation for this apparent inconsistency is that all previous studies focused on time estimation of short-term intervals, and here we have examined much longer intervals. Existing evidence suggests that time estimation in longer intervals is less affected by emotions than shorter intervals^45^. Finding no link between time perception in long intervals, anxiety and stress, supports our hypothesis that the causes of the observed time inflation are of cognitive rather than emotional source. Notwithstanding this, it is important to note that in this study emotion was measured at the time of testing, using questionnaires prompting participants to respond according to the preceding few weeks. Therefore, we cannot rule out the effect of emotional state at the time of encoding, and also not the effect of mood at the time of testing and its modulation by the presented events.

To conclude, in this article we showed evidence that the popular observation that time is “elastic” reflects the real experience during April of 2020—subjective time slowed-down. Moreover, temporal perception at that time was drastically affected by whether events were related or unrelated to the pandemic. This demonstrates that not only is our subjective time flexible and drastically affected by the circumstances, but also that it is affected by what we are attempting to estimate. There is no single timing mechanism but multiple scales by which time is estimated according to its contents and context.

## Supplementary Information

Supplementary Information.

## Data Availability

The data that support the findings of this study are available from the corresponding author, KT, upon reasonable request.
