# Supplementary material for: Evidence for content-dependent timing of real-life events during COVID-19 crisis
Source: Sci Rep. 2022 Jun 2;12:9220. doi: 10.1038/s41598-022-13076-6 (PMC9161651; doi:10.1038/s41598-022-13076-6)
Supplement: Supplementary file 1 — Supplementary Information. [file 41598_2022_13076_MOESM1_ESM.docx]

Evidence for content-dependent timing of real-life events during COVID-19 crisis

Keren Taub, Dekel Abeles & Shlomit Yuval-Greenberg

Supplementary 1: Full list of events in order of real occurrence (order was mixed during test)

The events presented here are translated from Hebrew.

| **Event** | **Group** | **Original date** | **Pair #** |
| --- | --- | --- | --- |
| Kobe Briant died in a helicopter crash | Non-COVID | 26/01/2020 | - |
| US president Donald Trump released the Israel-Palestine peace plan | Non-COVID | 28/01/2020 | 1 |
| Israel banned all flights from China | COVID | 30/01/2020 | 2 |
| Israeli Naama Issachar freed from the Russian prison | Non-COVID | 30/01/2020 | 2 |
| Israel issued travel warning for China and recommends all Israeli citizens currently in the country to return | COVID | 31/01/2020 | 1 |
| Passengers from the Diamond Princess Cruise Ship returned to Israel | COVID | 21/02/2020 | - |
| South Korean pilgrims who toured Israel were found to be infected with COVID | COVID | 22/02/2020 | - |
| The first COVID infection in Israel – a toy store manager - was reported | COVID | 27/02/2020 | - |
| Israel chose its representative song for Eurovision 2020 | Non-COVID | 03/03/2020 | 3 |
| More than a thousand high school students were quarantined after one student was infected with COVID | COVID | 04/03/2020 | 3 |
| The Israeli ministry of health published first instructions for quarantining travelers returning from France, Germany, Spain and Austria | COVID | 04/03/2020 | 4 |
| Results for the 23 Knesset elections were published | Non-COVID | 04/03/2020 | 5 |
| A man was arrested following the murder of his daughter and an attempt to murder his wife and second daughter | Non-COVID | 06/03/2020 | 4 |
| A general lockdown was announced in Italy | COVID | 07/03/2020 | 5 |
| Israeli prime minister announced quarantine for all international arrivals | COVID | 09/03/2020 | 6 |
| Knesset Member Orli Levi-Abeksis abandoned her partnership with the left-wing alliance | Non-COVID | 10/03/2020 | 6 |
| World Health Organization declared the coronavirus outbreak as a pandemic | COVID | 11/03/2020 | 7 |
| Israeli singer Eyal Golan got married | Non-COVID | 12/03/2020 | 7 |
| Prime minister announced that schools and universities should stop in-person learning and revert to distant learning | COVID | 12/03/2020 | 8 |
| The 23 Knesset was sworn | Non-COVID | 16/03/2020 | 8 |
| Benny Ganz received the mandate from the president to form a coalition | Non-COVID | 16/03/2020 | 9 |
| "Dan Panorama" hotel in Tel Aviv was turned into a quarantine facility for COVID patients | COVID | 17/03/2020 | 9 |
| Israeli government approved security service to use mobile surveillance to track COVID cases | COVID | 18/03/2020 | 10 |
| First Israeli died from the coronavirus | COVID | 20/03/2020 | - |
| Sixty-five Chabad followers were found positive for coronavirus after arriving from the US | COVID | 23/03/2020 | - |
| Israel supreme court forced Knesset chairman Yuli Adelstein to convene the Knesset | Non-COVID | 23/03/2020 | 10 |
| Britain changed its policy and announced a lockdown | COVID | 23/03/2020 | - |
| Tokyo 2020 Olympic games are canceled and postponed by one year | COVID | 24/03/2020 | - |
| The "Blue and White" political alliance splits | Non-COVID | 26/03/2020 | 11 |
| Britain prime minister Boris Johnson was tested positive for coronavirus | COVID | 27/03/2020 | 11 |
| Israeli prime minister is in quarantine following exposure to the coronavirus | COVID | 30/03/2020 | - |
| The Israeli minister of health was tested positive to coronavirus | COVID | 02/04/2020 | - |

Supplementary 2: Analysis of delay discounting

Delay discounting is the decline in value of a delayed, relative to an immediate, reward. In our preregistration document, we raised the hypothesis that inflated time estimation is related to the delay discounting effect, meaning that when the difference between the subjective and the objective time is larger, so is the difference in value of a delayed and an immediate reward. To examine this question, we have asked participants a theoretical question: for how much money, to be received in a year, they would be willing to give up an immediate reward of 1000 Shekels (based on Takahashi ,2004).

After removal of outliers (responses that were larger than median + 3 times the interquartile range) we analyzed the reports of 426 participants, but found no evidence for a correlation between the overall subjective time estimation and delay discounting (Pearson R=0.020, p=0.687). We further conducted a hierarchical linear regression analysis to test the contribution of three of the demographic factors (gender, age and age^2^), the three psychological factors (anxiety, stress, and COVID threat perception) and the overall time estimation error on the prediction of delay discounting. None of the factors was found to significantly predict delay discounting (age: β=-0.041, FDR corrected p=0.253; age^2^: β=-0.033, FDR corrected p=0.1.069; gender: β=0.098, FDR corrected p=0.308; anxiety: β=0.033, FDR corrected p=1.062; stress: β=0.036, FDR corrected p=1.367; COVID threat perception: β=0.029, FDR corrected p=0.2.026; overall time estimation error: β=0.004, FDR corrected p=0.929).
